# Beyond the Black Box: Is Artificial Intelligence Ready to Reshape Neurosurgical Decision‐Making? A Narrative Review

**DOI:** 10.1002/hsr2.73117

**Published:** 2026-08-26

**Authors:** Dip Bahadur Singh, Yashoda Dangi, Bhishma Prasad Pokharel

**Affiliations:** ^1^ Department of Health Informatics, School of Engineering Kathmandu University Dhulikhel Nepal; ^2^ Valley College of Technical Sciences Purbanchal University Biratnagar Nepal; ^3^ Province Hospital, Karnali Province Surkhet Birendranagar Nepal

**Keywords:** artificial intelligence, black box, clinical decision‐making, deep learning, digital health, ethics, explainable AI, implementation science, machine learning, neurosurgery

## Abstract

**Background and Aims:**

Artificial intelligence (AI) is increasingly being integrated into neurosurgical practice, offering capabilities in diagnostic imaging, surgical planning, and outcome prediction. However, the “black box” nature of many AI systems generating recommendations without a transparent rationale poses fundamental challenges to adoption in a specialty defined by high‐stakes, irreversible interventions. This review critically examines whether AI is ready to reshape neurosurgical decision‐making, synthesizing current evidence while systematically analyzing technical, ethical, and regulatory barriers to clinical integration.

**Methods:**

A systematic search of PubMed, Scopus, and Web of Science databases was conducted for peer‐reviewed studies published between January 2020 and March 2026. Articles reporting AI applications in neurosurgical diagnosis, prognosis, or intraoperative guidance were included. Data were synthesized thematically across clinical domains, with a focus on model interpretability, validation status, and implementation barriers.

**Results:**

AI demonstrates significant capabilities: diagnostic accuracy exceeding AUC 0.90 in tumor classification, prognostic improvements up to 15% over traditional methods, and 10%–20% complication reductions with AI‐assisted planning. Currently, FDA‐cleared tools enable automated tumor segmentation, aneurysm detection, and spinal navigation. However, critical gaps persist: external validation remains rare (< 20% of studies; e.g., 10 of 60 cerebrovascular studies (16.7%) reported external validation, with pooled AUC 0.84 [95% CI, 0.79–0.88] for thrombectomy outcome), most models are trained on homogeneous single‐center datasets, and the “black box” problem limits clinician trust. From an implementation science perspective, human factors, including workflow integration and cognitive load, remain underexplored.

**Conclusion:**

AI is not ready for independent decision‐making in neurosurgery but serves as a powerful augmentative tool when limitations are transparently addressed. Lessons from neurosurgery offer a blueprint for AI integration across high‐stake medical specialties. The black box must be opened before AI can truly reshape clinical practice.

## 1 Introduction

The operating room of the near future may look subtly different from today's: a neurosurgeon pauses before initiating a tumor resection, glancing at a monitor where an AI system has overlaid probabilistic margins onto the cortical surface, highlighting eloquent areas to preserve, and suggesting an optimal corticectomy trajectory. The algorithm analyzes thousands of similar cases, integrating multimodal imaging, intraoperative ultrasound, and real‐time fluorescence data. The surgeon nods, adjusts the plan slightly based on intraoperative findings, and then proceeds with the procedure. The AI did not make the decision, but it fundamentally reshaped the decision‐making process.

This vision encapsulates both the promise and tension surrounding artificial intelligence in neurosurgery. AI technologies have advanced remarkably. Machine learning (ML) and deep learning (DL) algorithms now achieve diagnostic accuracies that exceed those of expert clinicians in specific tasks [1]. FDA‐cleared tools for tumor segmentation, aneurysm detection, and spinal navigation demonstrate enhanced precision and reduced observer variability [2]. Prognostic models improve outcome predictions by up to 15% compared to traditional statistical methods [3]. Intraoperative AI applications, from instrument tracking to ultrasound analysis, are moving from benchtop to bedside [4].

However, a fundamental obstacle impedes clinical translation: the “black box” problem, defined here, in the neurosurgical context, as the inability of a clinician to inspect or reconstruct the internal decision logic by which an AI system arrives at a diagnostic, prognostic, or procedural recommendation, so that the recommendation cannot be independently verified against the anatomical and pathophysiological reasoning that governs surgical practice. Most contemporary AI systems, particularly deep learning networks, operate as opaque models. They generate outputs a diagnosis, a risk score, and a recommended trajectory without providing interpretable rationale for their conclusions [5]. A surgeon facing an AI recommendation cannot easily discern whether the algorithm considers relevant anatomical variants, whether its training data include similar patient populations, or whether subtle imaging features influence output. This opacity fundamentally clashes with neurosurgical practice, where decisions carry life‐altering consequences and clinicians bear ultimate accountability.

The question posed by this review is artificial intelligence ready to reshape neurosurgical decision‐making. Therefore, it requires a nuanced examination. Readiness is not merely a matter of technical performance metrics. It encompasses interpretability, generalizability, ethical soundness, regulatory alignment, and integration into the clinical workflow. This narrative review synthesizes current evidence across neurosurgical subspecialties, systematically analyzes barriers to adoption, and proposes a path forward for responsible AI integration.

## 2 Methods

### 2.1 Search Strategy and Information Sources

A systematic search of the literature was conducted in accordance with the Preferred Reporting Items for Systematic Reviews and Meta‐Analyses (PRISMA) guidelines, adapted for narrative synthesis [6]. Three electronic databases, PubMed, Scopus, and Web of Science, were searched for peer‐reviewed literature published between January 1, 2020, and March 1, 2026. The search strategy combined controlled vocabulary terms (MeSH and Emtree) and free‐text keywords related to three core concepts: artificial intelligence, neurosurgery, and clinical decision‐making, following established methods for AI literature reviews [7].

The complete search string for PubMed was:

((“artificial intelligence”[MeSH Terms] OR “artificial intelligence”[tiab] OR “machine learning”[MeSH Terms] OR “machine learning”[tiab] OR “deep learning”[tiab] OR “neural network”[tiab] OR “AI”[tiab] OR “ML”[tiab] OR “explainable AI”[tiab] OR “XAI”[tiab]) AND (“neurosurgery”[MeSH Terms] OR “neurosurgery”[tiab] OR “neurosurgical”[tiab] OR “brain tumor”[tiab] OR “brain tumour”[tiab] OR “glioma”[tiab] OR “meningioma”[tiab] OR “intracranial aneurysm”[tiab] OR “cerebrovascular”[tiab] OR “epilepsy surgery”[tiab] OR “deep brain stimulation”[tiab] OR “spinal surgery”[tiab] OR “neurotrauma”[tiab]) AND (“decision making”[MeSH Terms] OR “decision making”[tiab] OR “clinical decision”[tiab] OR “diagnosis”[tiab] OR “prognosis”[tiab] OR “treatment planning”[tiab] OR “surgical planning”[tiab] OR “outcome prediction”[tiab]))

Analogous search strings, adapted to the syntax of each database (Scopus TITLE‐ABS‐KEY and Web of Science TS field searches), were applied to Scopus and Web of Science; the complete strings for all three databases are reproduced verbatim in Supplementary Appendix A for transparency and reproducibility. The search was limited to English‐language publications. Conference proceedings and preprint articles were included if they presented the original data and had undergone a peer review.

### 2.2 Eligibility Criteria

Studies were included if they: (1) reported original research (including retrospective cohorts, prospective studies, randomized controlled trials, or conference proceedings with original data); (2) described systematic reviews or meta‐analyses of AI applications; (3) focused on AI, machine learning, or deep learning applications in neurosurgical diagnosis, prognosis, intraoperative guidance, or perioperative decision‐making; and (4) reported quantitative performance metrics or qualitative implementation findings.

Studies were excluded if they: (1) were opinion pieces, editorials, or commentaries without original data; (2) described purely preclinical or ex vivo models without clinical validation; (3) focused on non‐neurosurgical applications; and (4) were duplicate publications.

### 2.3 Study Selection and Data Extraction

Titles and abstracts were screened independently by two reviewers (D.B.S. and Y.D.) against the eligibility criteria. The full texts of potentially eligible articles were retrieved and assessed independently. Disagreements were resolved by consensus or consultation with a third reviewer (B.P.). Data were extracted into a standardized form capturing: (1) study characteristics (author, year, country, study design, sample size); (2) AI model details (algorithm type, training data source, interpretability features); (3) clinical application domain (diagnostic, prognostic, intraoperative, spinal, vascular, oncological, functional, trauma); (4) performance metrics (accuracy, AUC, sensitivity, specificity, calibration); (5) validation status (internal cross‐validation, temporal validation, external validation on independent dataset) [8]; (6) reported implementation barriers or ethical considerations.

### 2.4 Data Synthesis

Given the heterogeneity of the studies, a narrative synthesis approach was adopted. Findings were organized thematically according to the clinical application domain (diagnostic, prognostic, intraoperative) and neurosurgical subspecialty (neuro‐oncology, vascular, functional, spinal, and trauma). A critical appraisal framework was applied to assess evidence quality, with particular attention paid to validation methodology, risk of bias, and reporting transparency [7]. Quantitative performance summaries were generated where sufficient homogeneous data existed; otherwise, ranges and illustrative examples were presented.

### 2.5 Statistical Analysis and Reporting

As a narrative synthesis of previously published, aggregate‐level data rather than a primary study or meta‐analysis, this review did not generate a new patient‐level dataset and therefore did not perform inferential hypothesis testing of its own; no statistical software package was used to derive novel *p* values. Reporting of quantitative findings extracted from the included studies followed the recommendations of Assel et al. [9] for the reporting of statistics in clinical research, adapted for narrative synthesis. Where the original studies reported measures of precision, these are reproduced here as 95% confidence intervals (CIs); where an included study reported a mean and standard deviation (SD) for normally distributed data or a median and interquartile range (IQR)/range for non‐normally distributed data, that convention was preserved rather than converted. Proportions and percentages are accompanied by their originating numerator and denominator whenever these were reported in the source publication; for a minority of older narrative reviews cited here, the primary studies did not disclose exact counts, and this is noted as a limitation in Section 10. Any *p* values quoted from source studies are reported using standard convention (*p* < 0.001; to the nearest thousandth for 0.001–0.01; to the nearest hundredth for ≥ 0.01; *p * > 0.99 where applicable) and are never presented without the accompanying effect estimate and statistical test from which they derive. The critical appraisal applied to extracted evidence (Section 2.4) followed AMSTAR‐2 and PRISMA‐based domains for study design, risk of bias, and reporting transparency; no a prior significance threshold was set for this narrative synthesis because no new hypothesis test was conducted, and all quantitative syntheses reported here (e.g., Table 1) are explicitly exploratory descriptive summaries of previously published, prespecified analyses conducted by the original study authors rather than prespecified analyses of this review. Prior to inclusion, all candidate references were screened against the Retraction Watch database and PubMed's retraction tags, and none of the cited articles had been retracted or formally corrected at the time of the (March 2026) search.

**Table 1:** Summary of AI performance metrics across neurosurgical application domains, with source study denominators.

Domain	Performance metric	Comparison
Diagnostic accuracy	AUC > 0.90	Superior to clinical diagnosis in 41.3% of comparisons [1]
Prognostic improvement	Up to 15%	Versus traditional statistical methods [3]
Complication reduction	10%–20%	With AI‐assisted surgical planning [10]
Tumor classification	> 96% accuracy	Deep learning on MRI data [11]
Seizure freedom prediction	AUC up to 0.95	Multimodal models in epilepsy surgery [12]

*Note:* Values represent point estimates as reported in the cited primary or secondary sources, extracted narratively rather than pooled by this review; 95% confidence intervals and source denominators, where available, are reported in the accompanying text.

Abbreviations: AUC, area under the receiver operating characteristic curve; AI, artificial intelligence.

## 3 Current Capabilities: What AI Can Already Do

### 3.1 Diagnostic Applications

AI's most mature applications of AI in neurosurgery lie in diagnostic imaging. Deep learning models applied to MRI, CT, and ultrasonography consistently achieve performance comparable or superior to expert clinicians in specific tasks [1].

#### 3.1.1 Neuro‐Oncology

Convolutional neural networks demonstrate > 96% accuracy in distinguishing tumor types from MRI data [11]. AI‐based segmentation tools enable automated volumetric assessment of gliomas, meningiomas, and metastases, reducing inter‐observer variability and supporting more precise surgical planning [13]. Stimulated Raman histology combined with AI allows rapid intraoperative identification of tumor margins, achieving diagnostic accuracy exceeding conventional frozen section analysis in less than 150s [14]. Topic modeling algorithms, such as latent dirichlet allocation (LDA), facilitate non‐invasive tumor grading by linking imaging features with molecular and genetic profiles.

#### 3.1.2 Vascular Neurosurgery

FDA‐cleared platforms enable automated aneurysm detection and characterization. Machine learning algorithms analyze CT angiography and digital subtraction angiography to identify aneurysms, classify them by size, shape, and location, and stratify rupture risk [15]. Deep learning models for intracranial hemorrhage detection on head CT demonstrate high sensitivity and specificity, potentially expediting diagnosis in emergency settings [16].

#### 3.1.3 Functional Neurosurgery

AI assists in localizing epileptogenic zones by integrating electroencephalogram (EEG) and neuroimaging data. Multimodal machine learning models incorporating clinical variables, EEG findings, and imaging achieve area under the curve (AUC) values up to 0.95 for predicting seizure freedom after epilepsy surgery [12]. For deep brain stimulation (DBS) in Parkinson's disease, machine learning algorithms analyze functional MRI and clinical data to predict optimal stimulation parameters, potentially reducing the time‐consuming trial‐and‐error process of programming.

### 3.2 Prognostic and Predictive Applications

Beyond diagnosis, AI demonstrates particular strength in integrating diverse data types to generate personalized prognostic estimates, a task that challenges human cognition owing to the volume and complexity of information.

A systematic review of cerebrovascular applications found that machine learning models achieved pooled AUCs of 0.84 for functional outcome after mechanical thrombectomy and 0.89 for outcomes after subarachnoid hemorrhage [3]. Among studies comparing ML with traditional statistical models, ML was superior in 78.6% of cases [17]. For unruptured aneurysm management, AI‐based rupture risk prediction incorporates not only aneurysm morphology but also hemodynamic factors and patient‐specific characteristics, supporting more informed decisions about intervention versus observation [15].

#### 3.2.1 Spinal Neurosurgery

Machine learning models predict hospital length of stay, discharge disposition, and complication risks based on preoperative and intraoperative data [18]. AI‐assisted gait analysis refines the diagnosis of spinal‐related movement disorders, while predictive algorithms help surgeons anticipate unfavorable treatment outcomes and adjust strategies accordingly [2].

#### 3.2.2 Neurotrauma

AI models predict the need for emergency neurosurgical intervention within 24 h of moderate‐to‐severe traumatic brain injury [18]. Deep learning‐based intracranial pressure estimation using noninvasive imaging and clinical data may reduce the need for invasive monitoring in selected patients [4].

### 3.3 Intraoperative Applications

The intraoperative environment represents a frontier for AI integration, although evidence remains less mature than for diagnostic applications [19].

#### 3.3.1 Surgical Navigation

Enhanced deep learning improves anatomical structure visualization and recognition compared to conventional methods [4]. AI algorithms register preoperative imaging to intraoperative anatomy, compensating for brain shifts, and enabling more precise targeting.

#### 3.3.2 Instrument Tracking

Using computer vision enables real‐time identification of surgical tools, supporting context‐aware warnings, and automated documentation [20].

#### 3.3.3 Video Segmentation

Algorithms label operative phases, track tissue planes, and may eventually provide real‐time guidance on resection boundaries [21].

#### 3.3.4 Intraoperative Ultrasound Analysis

Augmented by deep learning has been demonstrated in pilot human studies, with algorithms detecting foreign bodies and delineating tumor margins [22]. Intraoperative MRI reconstruction accelerated by deep learning may shorten workflow delays in iMRI‐equipped suites [23].

#### 3.3.5 Robotic Systems

Including ROSA and Mazor X, integrated with AI algorithms, provide real‐time intraoperative assistance, improving trajectory planning and targeting precision in stereotactic and spinal procedures [24].

### 3.4 Quantitative Performance Summary

Across applications, AI demonstrates consistent performance advantages (Table 1):

To illustrate these summary figures with the precision recommended above, the largest contributing cerebrovascular systematic review (60 studies; 71 predicted outcomes) reported a pooled AUC of 0.84 (95% CI, 0.79–0.88; *n* = 32 stroke studies) for functional outcome after mechanical thrombectomy, and pooled AUCs of 0.89 (95% CI, 0.76–0.95) and 0.90 (95% CI, 0.66–0.98) for functional outcome and delayed cerebral ischemia, respectively, after subarachnoid hemorrhage (*n* = 16 studies). In that same review, machine learning outperformed standard statistical models in 33 of 42 studies (78.6%) and outperformed non‐machine‐learning clinical prediction models in 9 of 10 studies (90%), while only 10 of 60 studies (16.7%) reported external validation and 40 of 60 (66.7%) relied on single‐institution cohorts. Comparable numerator/denominator detail was not uniformly reported across the other secondary sources synthesized in this narrative review; this heterogeneity in the granularity of the underlying literature is acknowledged as a limitation in Section 10.

## 4 The Black Box Problem: Why Opacity Matters

### 4.1 Defining the Black Box

The term “black box” in AI refers to systems whose internal workings are not transparently accessible to human understanding. Deep neural network learning to classify brain tumors may contain millions of parameters adjusted through iterative training; the final model represents a complex web of weighted connections that defies simple interpretation. When such a model recommends a diagnosis, it cannot easily explain *which* imaging features drove its conclusion, *how* it weighed conflicting evidence, or *whether* its reasoning aligns with the established anatomical and pathological principles [5].

This opacity contrasts sharply with traditional clinical decision‐making. When a neurosurgeon recommends against operating on a posterior circulation aneurysm, they can articulate their reasoning: the patient's age and comorbidities, aneurysm morphology and location, estimated rupture risk, and surgical morbidity data from comparable cases. The patient and family can question, challenge, and ultimately decide whether to accept the recommendations. An AI system that offers the same conclusion without rationale cannot participate in this shared decision‐making process.

### 4.2 Why Interpretability Matters in Neurosurgery

Several factors render the black‐box problem particularly acute in neurosurgery.

#### 4.2.1 High‐Stakes Consequences

Neurosurgical decisions carry the potential for irreversible harm, permanent neurological deficits, cognitive impairment, or death. Errors arising from AI recommendations have different moral and legal implications than those in purely algorithmic domains. When a black‐box model errs, determining why it erred, and thus preventing future similar errors becomes extraordinarily difficult [25].

#### 4.2.2 Clinical Nuance

Neurosurgical decision‐making requires integrating factors that may not be fully captured in training data: subtle variations in anatomy, patient preferences, institutional capabilities, and dynamic intraoperative findings. A black‐box model cannot explain whether it has accounted for such nuances, leaving clinicians uncertain when to trust versus override its recommendations [19].

#### 4.2.3 Legal Liability

When decisions influenced by AI lead to adverse outcomes, questions of responsibility arise. Is the clinician liable to follow an opaque recommendation? The hospital that implemented the system: The developer for creating it? Current medicolegal frameworks offer limited guidance, and black‐box models compound this uncertainty [26].

#### 4.2.4 Informed Consent

Patients have the right to understand how their care decisions are made. If AI significantly influences surgical recommendations, can informed consent be genuinely obtained without explaining its role and limitations? The opacity of black‐box systems challenges this fundamental ethical requirement [27].

#### 4.2.5 Trust Calibration

Clinicians must calibrate their trust in AI systems knowing when to rely on recommendations and when to override them. This calibration is impossible without understanding the model's reasoning, failure modes, and the boundaries of its competence [28].

### 4.3 Consequences of Opacity in Practice

The black‐box problem manifests as tangible clinical concerns. A recent letter to the editor highlighted that “most neurosurgical AI tools still operate as black‐box models, and a tension persists between clinical expectations for interpretability and the limited transparency of current systems“ [29]. This tension has the following practical implications:

#### 4.3.1 Risk of Hidden Errors

Black‐box models may learn spurious correlations associating imaging artifacts with pathology or institutional labels with outcomes without clinicians being able to detect such flawed reasoning [30].

#### 4.3.2 Difficulty Troubleshooting

When a model performs poorly on specific patient subgroups, understanding *why* it requires access to its internal representations, which black‐box systems do not provide [31].

#### 4.3.3 Regulatory Hurdles

The FDA increasingly requires the explainability of AI‐driven tools used in high‐risk medical decisions, recognizing that clinicians cannot safely use systems whose reasoning cannot be interrogated [8].

#### 4.3.4 Clinician Reluctance

Surveys suggest that surgeons remain hesitant to adopt AI tools whose recommendations cannot be explained, particularly in intraoperative contexts where real‐time decisions carry immediate consequences [32].

## 5 Beyond the Black Box: Barriers to Clinical Adoption

### 5.1 Limited External Validation

A striking finding across systematic reviews is the paucity of external validation. Among cerebrovascular AI studies, only 16.7% performed validation using independent datasets [3]. Among epilepsy surgery ML models, cross‐validation predominated, with only two studies undertaking prospective validation [12]. Most cohorts were derived from single institutions (66.7% of cerebrovascular studies), limiting generalizability [17].

This is important because model performance often degrades when applied to new populations, imaging protocols, or clinical settings. A tumor segmentation model trained on high‐resolution 3 T MRI from a tertiary academic center may fail when deployed at a community hospital using 1.5 T scanners. An outcome prediction model developed for patients from one geographic region may not generalize to populations with different genetic backgrounds, environmental exposures, or healthcare access patterns.

The rarity of external validation means that published performance metrics are likely to overestimate the real‐world accuracy. As one critical appraisal noted, “it is not uncommon to overfit the models, and performance tends to decrease when the environment in which they are tested differs“ [33].

### 5.2 Algorithmic Bias and Health Disparities

AI models learn from training data; if the data lack diversity, the resulting algorithms may perform poorly or dangerously for underrepresented populations. This concern is particularly acute in neurosurgery, where race, ethnicity, socioeconomic status, and geography influence disease presentation, treatment access, and outcomes [34].

A machine learning model trained predominantly on male patients may introduce bias against female patients [35]. Algorithms developed on datasets lacking racial diversity may misclassify pathology or miscalculate the risk for minority populations [36]. Models optimized in high‐resource settings may fail when deployed in rural or low‐income environments with different imaging equipment, clinical workflows, and patient populations [37].

These consequences extend beyond the technical performance. Biased algorithms could exacerbate existing healthcare disparities, leading to delayed diagnosis, inappropriate treatment recommendations, or inaccurate prognostic counseling for already marginalized groups [38]. Addressing this requires intentional efforts to build inclusive datasets, validate models across diverse populations, and monitor disparate performance after deployment.

### 5.3 Data Privacy and Security

Neurosurgical AI applications require access to highly sensitive data, such as neuroimaging revealing brain structure, EEG recordings capturing neural activity, cognitive assessments, and detailed clinical histories. This information is not merely personal, but fundamentally constitutive of identity and cognition [39].

The aggregation and analysis of such data raises privacy concerns. De‐identification may prove insufficient for neuroimaging data, which potentially contain sufficient structural information to re‐identify individuals. Brain‐computer interface data could theoretically reveal thoughts, intentions, or mental states. Compliance with regulations such as HIPAA and GDPR is necessary, but not sufficient; ethical data governance requires ongoing attention to evolving privacy risks.

### 5.4 Accountability and Liability

When AI‐assisted decisions result in adverse outcomes, responsibility assignment becomes complex. Three recurring scenarios illustrate this difficulty and remain unresolved under current frameworks. First, when a surgeon follows an AI recommendation that proves incorrect and causes harm, it is unclear whether liability rests with the surgeon who relied on the system, the hospital that implemented it, or the developer that created the flawed model. Second, when an AI system fails to flag an impending complication, responsibility for that missed warning is similarly undefined. Third, when a model's performance degrades over time because of shifting patient populations or imaging protocols, and an adverse event occurs before the drift is detected, no established framework assigns responsibility for ongoing post‐deployment monitoring.

Current legal frameworks provide limited guidance. Recent FDA, EU, and WHO guidelines emphasize lifecycle monitoring, transparency, and real‐world evidence; however, these regulatory requirements have not yet translated into clear liability standards [26]. Until accountability is clarified, clinicians and institutions may hesitate to adopt AI tools; however, this is promising.

### 5.5 De‐Skilling and Autonomy Erosion

A less‐discussed but equally important concern involves the potential for AI to erode clinical skills and professional autonomy. As surgeons increasingly rely on AI‐generated recommendations, they may lose the ability to make equivalent judgments independently [40].

This “de‐skilling” process has precedents in other domains, where automation transformed human expertise. Pilots who rely heavily on autopilots may struggle to fly manually under unexpected conditions. Similarly, neurosurgeons who depend on AI for tumor segmentation may find atrophy in their interpretive skills. When AI systems fail, surgeons must be capable of stepping in with fully functional clinical judgment.

Preserving autonomy requires deliberate approaches to AI integration: systems should augment rather than replace human decision‐making, training should maintain emphasis on foundational skills, and clinicians should understand AI limitations sufficiently to know when to override recommendations [41].

### 5.6 Regulatory and Infrastructure Challenges

The regulatory landscape of AI in medicine is evolving rapidly. The FDA's Software as a Medical Device Guidance emphasizes lifecycle monitoring and risk‐based review [8]. The EU AI Act (2024) classifies intraoperative AI as high‐risk, requiring strict audit trials, documented risk management, and human oversight [42]. The WHO's 2024 guidance recommends continuous real‐world evidence after the market launch [43].

Meeting these requirements requires infrastructure that many healthcare settings lack: computational resources for model deployment, technical expertise for integration and monitoring, and robust data‐governance frameworks. In low‐ and middle‐income countries, where neurosurgical capacity is often most strained, these requirements may be prohibitive [44]. Paradoxically, the populations that might benefit the most from AI assistance may be the least able to access it under current regulatory and infrastructure models.

## 6 Opening the Black Box: Paths to Transparency

### 6.1 Technical Approaches to Explainability

Several technical strategies can render AI systems more interpretable.

#### 6.1.1 Saliency Mapping

Identifies which regions of an input image most influenced the model's output. For a tumor classification algorithm, saliency maps highlight pixels that drive the diagnosis, allowing clinicians to verify that the model attended to relevant pathology rather than artifacts [45].

#### 6.1.2 Attention Mechanisms

In neural networks reveal which parts of the input the model “focuses on” when generating predictions. In multimodal models integrating clinical data, EEG, and imaging, attention weights can indicate which data sources contribute the most to the output [46].

#### 6.1.3 Concept‐Based Explanations

Map model representations onto human‐understandable concepts, rather than reporting that a particular combination of pixels triggered a classification, concept‐based methods might indicate that the model detected features consistent with “necrosis,” “enhancement,” or “mass effect“ [47].

#### 6.1.4 Surrogate Models

Approximate complex black‐box systems with simpler locally interpretable models. Although the global behavior of a deep network may be inscrutable, its predictions for a specific case can be approximated by a decision tree or linear model that provides a transparent rationale [6].

#### 6.1.5 Counterfactual Explanations

Show how changing input features would alter the model's output. For a patient predicted to have poor surgical outcome, a counterfactual explanation might indicate: “If the patient's age were 10 years younger, the predicted outcome would be favorable.” This helps clinicians understand which factors most influence the prediction [48].

### 6.2 User‐Centered Design for Explainability

Technical explainability must be paired with a user‐centered design. Explanations are only valuable if they meet clinicians’ needs in real‐world contexts [49]. This requires:

#### 6.2.1 Stakeholder Engagement

Involving neurosurgeons in designing explanation interfaces ensures that the outputs align with the clinical reasoning patterns and information needs.

#### 6.2.2 Task‐Specific Explanations

The type of explanation useful for preoperative planning (e.g., feature importance) may differ from that needed intraoperatively (e.g., real‐time saliency overlay).

#### 6.2.3 Cognitive Load Considerations

Explanations must be concise and intuitive to avoid information overload during high‐stake decision‐making.

#### 6.2.4 Trust Calibration Feedback

Systems should provide sufficient information for clinicians to calibrate their trust appropriately, including confidence estimates and known limitations.

### 6.3 Regulatory and Reporting Standards

Regulatory bodies are increasingly mandating explainability of high‐risk AI applications. The FDA requires that AI‐driven tools for tumor segmentation, hemorrhage detection, or operative planning “be explainable so that surgeons can interpret the rationale behind system recommendations” [8].

Reporting guidelines such as Transparency in the Reporting of Artificial Intelligence (TITAN) promote standardized disclosure of model characteristics, training data, validation approaches, and interpretability features [50]. Adherence to such guidelines facilitates critical appraisal and enables clinicians to make informed judgments about the suitability of AI tools for their practice.

## 7 The Path Forward: Recommendations for Responsible Integration

### 7.1 For Researchers and Developers

#### 7.1.1 Prioritize External Validation

Models should be tested on independent multi‐institutional datasets before clinical deployment. Performance degradation across settings must be quantified and reported [33].

#### 7.1.2 Embrace Explainable AI

Interpretability should be a design requirement and not an afterthought. Models should provide clinically meaningful explanations for their outputs [51].

#### 7.1.3 Build Diverse Datasets

Training data must represent the full spectrum of patient populations, imaging protocols, and clinical settings in which the models will be deployed. International collaboration can facilitate dataset diversity [38].

#### 7.1.4 Report Failure Modes Transparently

Publications should describe not only the average performance, but also the conditions under which models fail. This enables clinicians to understand the limitations and avoid inappropriate applications [7].

#### 7.1.5 Conduct Prospective Trials

The field requires randomized controlled trials and prospective cohort studies measuring impact of AI's on clinical decisions, patient outcomes, workflow efficiency, and healthcare costs [52].

### 7.2 For Clinicians and Healthcare Institutions

#### 7.2.1 Develop AI Competency

Neurosurgical training should include education on AI principles, interpretability, limitations, and ethical considerations. Surgeons must understand these tools to use them responsibly [53].

#### 7.2.2 Establish Oversight Mechanisms

Institutions should create multidisciplinary committees to evaluate AI tools before adoption, monitor performance after deployment, and investigate adverse events potentially related to AI [54].

#### 7.2.3 Maintain Human‐Centered Decision‐Making

AI should augment, not replace, clinical judgment. Surgeons must retain the final authority and be prepared to override recommendations when appropriate [41].

#### 7.2.4 Advocating for Equitable Access

Institutions should work to ensure that the benefits of AI extend to underserved populations, avoiding a two‐tiered system where advanced tools are concentrated in well‐resourced settings [44].

### 7.3 For Regulators and Policymakers

#### 7.3.1 Clarifying Liability Frameworks

Clear guidance on responsibility for AI‐influenced decisions is essential for clinical adoption. This requires input from clinicians, developers, legal experts, and patient representatives [26].

#### 7.3.2 Mandate Lifecycle Monitoring

Regulatory approval should not be a one‐time event. Ongoing surveillance for performance drift, emerging biases, and unexpected failure modes is essential [43].

#### 7.3.3 Promote Interoperability

Standards for data exchange, model interfaces, and electronic health record integration would accelerate responsible adoption and reduce implementation barriers [55].

#### 7.3.4 Support Global Equity

Policies should facilitate AI access in low‐resource settings, potentially through tiered regulatory requirements, open‐source models, and technology transfer mechanisms [56].

## 8 Cross‐Specialty Generalizability and Implementation Science Perspectives

The challenges and solutions identified in neurosurgical AI are directly relevant to other high‐stakes medical domains. The problem of anatomical deformation during surgery, exemplified by “brain shift” in neurosurgery, has direct analogs in thoracic surgery (lung deformation during resection), abdominal surgery (liver shift during hepatectomy), and cardiac surgery (cardiac motion during valve procedures). AI models that compensate for such deformations in one speciality may inform adaptive algorithms across multiple surgical disciplines [24]. Similarly, the liability and informed consent questions raised by black‐box AI in neurosurgery provide a blueprint for medicolegal discussions that will emerge throughout medicine as AI tools proliferate [25]. Interoperability requirements for cross‐speciality deployment standards for data exchange, model interfaces, and electronic health record integration are essential to enable this knowledge transfer [55].

From an implementation science perspective, several underexplored human factors require further attention. The Non‐adoption, Abandonment, Scale‐up, Spread, Sustainability (NASSS) framework offers a useful lens for understanding AI adoption in neurosurgery [40]. Key questions include:

Several of these domains raise questions that remain largely unanswered. On the *condition/illness* dimension, it is unclear how AI alters the perceived complexity of neurosurgical conditions for the clinicians who use it. On the *technology* dimension, the interoperability requirements for AI tools to integrate with existing electronic health records, PACS, and intraoperative navigation platforms remain poorly defined. On the *value proposition* dimension, it is not yet established whether AI tools demonstrably reduce cognitive load or instead add complexity through alert fatigue and information overload. On the *adopter system* dimension, surgical trainees and attending surgeons likely interact differently with AI recommendations, and whether such use accelerates or attenuates skill development is unknown. On the *organization* dimension, the computational resources, technical support, and governance committees that hospitals must establish to deploy AI safely are not standardized. Finally, on the *wider system* dimension, payment models, malpractice insurance, and regulatory requirements together shape institutions’ willingness to adopt AI.

However, studies on workflow integration remain scarce. Preliminary evidence suggests that AI tools that add more than 2–3 min to the operative time face resistance from surgeons, while those requiring dedicated technical personnel may be economically unviable outside tertiary centers [30]. The IDEAL framework for surgical innovation provides guidance for evaluating integration challenges across procedural specialties [19]. User‐centered design research, involving neurosurgeons in interface development from inception, is essential to ensure that AI explanations align with clinical reasoning patterns and are presented at the appropriate cognitive load, particularly during intraoperative decision‐making, where attention is already taxed [49]. Understanding how machine learning recommendations influence clinician treatment selection is critical for designing effective decision support [28].

## 9 Conclusion

### 9.1 Ready to Reshape

Is artificial intelligence ready to reshape neurosurgical decision making? The evidence reviewed here supports a nuanced answer: yes, but with essential caveats.

AI is ready to reshape decision‐making because its capabilities have matured sufficiently to provide meaningful clinical support. The diagnostic accuracy of AI exceeds that of humans in specific tasks. Prognostic models integrate complex data to generate personalized estimates that inform counseling and planning for patients. Intraoperative applications enhance navigation, visualization, and real‐time guidance. When deployed appropriately, these tools can improve patient care.

### 9.2 Not Ready to Replace

AI is not ready to replace clinical judgment, operate without transparent rationale, or be deployed without rigorous validation and oversight. The black box must be opened before these systems can be trusted to make high‐stakes neurosurgical decisions. External validation must become the standard, not the exception. Diverse datasets must ensure equitable performance across all populations. Regulatory frameworks must clarify accountability and mandate life‐cycle monitoring. Clinicians must maintain their skills and judgment, using AI as an augmentative tool rather than as a crutch.

The lessons learned from neurosurgery extend far beyond this single speciality. The challenges of anatomical deformation, real‐time decision‐making, and irreversible consequences are shared across cardiothoracic surgery, interventional radiology, critical care medicine, and oncology. The frameworks for explainability, the imperative for external validation, and the human factor considerations discussed here offer a blueprint for AI integration in high‐stakes medicine. Implementation science must take its place alongside technical development, ensuring that AI tools are not only accurate but also usable, sustainable, and equitable across diverse healthcare settings.

The operating room of the near future will likely feature AI assistance, but the surgeon will remain in control, interpreting AI recommendations through the lens of experience, intuition, and patient‐centered values. The black box will not be eliminated entirely, but it will be rendered sufficiently transparent for responsible usage. And the fundamental question will shift from “Can AI make this decision?” to “How can AI help the surgeon make a better decision?”

This is the path forward: not artificial intelligence replacing human intelligence but artificial intelligence augmenting neurosurgical expertise. The black box must be opened and not discarded. The surgeon must remain the ultimate decision‐maker, equipped with better tools, but never supplanted by them. In this vision, AI truly reshapes neurosurgical decision‐making for the better.

## Author Contributions

**Dip Bahadur Singh:** conceptualization, investigation, funding acquisition, writing – original draft, methodology, validation, visualization, software, writing – review and editing, formal analysis, project administration, data curation, resources. **Yashod Dangi:** writing – original draft, writing – review and editing, project administration, resources. **Bhishma Prasad Pokharel:** conceptualization, validation, supervision, writing – review and editing.

## Funding

The authors have nothing to report.

## Ethics Statement

The authors have nothing to report. This study is a Narrative review based entirely on published literature and does not involve human participants or animals.

## Conflicts of Interest

The authors declare no conflicts of interest.

## Transparency Statement

Dip Bahadur Singh, the lead author and manuscript guarantor, affirms that this manuscript is an honest, accurate, and transparent account of the study being reported; that no important aspects of the study have been omitted; and that any discrepancies from the study as planned have been explained. This narrative review was conducted between January and March 2026 and was not prospectively registered, as no protocol registry currently accommodates narrative (as opposed to systematic) reviews; this is noted as a limitation in Section 10.

## Supporting information


Supporting File

## Data Availability

All relevant data are in the paper, and any query regarding the findings of this study can be obtained from online protal.
