# Supplementary material for: Beyond the Black Box: Is Artificial Intelligence Ready to Reshape Neurosurgical Decision‐Making? A Narrative Review
Source: Health Sci Rep. 2026 Aug 26;9(9):e73117. doi: 10.1002/hsr2.73117 (PMC13509019; doi:10.1002/hsr2.73117)
Supplement: Supplementary file 1 [file HSR2-9-e73117-s001.docx]

**SUPPLEMENTARY APPENDIX A: FULL DATABASE SEARCH STRINGS**

**PubMed:** (("artificial intelligence"[MeSH Terms] OR "artificial intelligence"[tiab] OR "machine learning"[MeSH Terms] OR "machine learning"[tiab] OR "deep learning"[tiab] OR "neural network"[tiab] OR "AI"[tiab] OR "ML"[tiab] OR "explainable AI"[tiab] OR "XAI"[tiab]) AND ("neurosurgery"[MeSH Terms] OR "neurosurgery"[tiab] OR "neurosurgical"[tiab] OR "brain tumor"[tiab] OR "brain tumour"[tiab] OR "glioma"[tiab] OR "meningioma"[tiab] OR "intracranial aneurysm"[tiab] OR "cerebrovascular"[tiab] OR "epilepsy surgery"[tiab] OR "deep brain stimulation"[tiab] OR "spinal surgery"[tiab] OR "neurotrauma"[tiab]) AND ("decision making"[MeSH Terms] OR "decision making"[tiab] OR "clinical decision"[tiab] OR "diagnosis"[tiab] OR "prognosis"[tiab] OR "treatment planning"[tiab] OR "surgical planning"[tiab] OR "outcome prediction"[tiab])) AND ("2020/01/01"[Date - Publication] : "2026/03/01"[Date - Publication]) AND English[Language]

**Scopus (TITLE-ABS-KEY):** TITLE-ABS-KEY(("artificial intelligence" OR "machine learning" OR "deep learning" OR "neural network" OR "AI" OR "ML" OR "explainable AI" OR "XAI") AND ("neurosurgery" OR "neurosurgical" OR "brain tumor" OR "brain tumour" OR "glioma" OR "meningioma" OR "intracranial aneurysm" OR "cerebrovascular" OR "epilepsy surgery" OR "deep brain stimulation" OR "spinal surgery" OR "neurotrauma") AND ("decision making" OR "clinical decision" OR "diagnosis" OR "prognosis" OR "treatment planning" OR "surgical planning" OR "outcome prediction")) AND PUBYEAR > 2019 AND PUBYEAR < 2027 AND LANGUAGE(English)

**Web of Science (Topic, TS=):** TS=("artificial intelligence" OR "machine learning" OR "deep learning" OR "neural network" OR "AI" OR "ML" OR "explainable AI" OR "XAI") AND TS=("neurosurgery" OR "neurosurgical" OR "brain tumor" OR "brain tumour" OR "glioma" OR "meningioma" OR "intracranial aneurysm" OR "cerebrovascular" OR "epilepsy surgery" OR "deep brain stimulation" OR "spinal surgery" OR "neurotrauma") AND TS=("decision making" OR "clinical decision" OR "diagnosis" OR "prognosis" OR "treatment planning" OR "surgical planning" OR "outcome prediction"); refined by Language: English and Timespan: 2020-01-01 to 2026-03-01.
